# Image-guided adaptive interventional radiotherapy (modern brachytherapy) boost for vaginal recurrences

**DOI:** 10.1007/s11547-026-02235-8

**Published:** 2026-06-20

**Authors:** Valentina Lancellotta, Maria Concetta La Milia, Enrico Rosa, Pierpaolo Dragonetti, Bruno Fionda, Leonardo Bannoni, Rosa Autorino, Alessia Nardangeli, Francesca Tortoreto, Benedetta Gui, Luca Russo, Angeles Rovirosa, Alessio Giuseppe Morganti, Gabriella Macchia, Maria Antonietta Gambacorta, Luca Tagliaferri

**Affiliations:** 1 Radioterapia Oncologica, Dipartimento di Diagnostica per Immagini e Radioterapia Oncologia, Fondazione Policlinico Universitario “A. Gemelli” IRCCS, 00168 Rome, Italy; 2 UOC Fisica per le Scienze della Vita, Dipartimento di Diagnostica per Immagini e Radioterapia Oncologica, Fondazione Policlinico Universitario A. Gemelli IRCCS, 00168 Rome, Italy; 3 Department of Theoretical and Applied Sciences, eCampus University, 22060 Novedrate, Italy; 4 Department of Radiation Oncology, IRCCS Regina Elena National Cancer Institute, Rome, Italy; 5 UOC di Radioterapia, Ospedale Isola Tiberina – Gemelli Isola, Rome, Italy; 6 Dipartimento Diagnostica per Immagini e Radioterapia Oncologica, Fondazione Policlinico Universitario A. Gemelli IRCCS, Rome, Italy; 7 Dipartimento Universitario di Scienze Radiologiche ed Ematologiche, Università Cattolica del Sacro Cuore, Rome, Italy; 8 Radiation Oncology Department, Hospital Clínic-Universitat de Barcelona, Barcelona, Spain; 9 Radiation Oncology, IRCCS Azienda Ospedaliero-Universitaria di Bologna, Bologna, Italy; 10 Radiation Oncology, Department of Medical and Surgical Sciences (DIMEC), University of Bologna, Bologna, Italy; 11 Radiation Oncology Unit, Responsible Research Hospital-Campobasso and Catholic University of Sacred Heart-Rome, Rome, Italy; 12 Università Cattolica del Sacro Cuore Sede di Roma, 00168 Rome, Italy

**Keywords:** Vaginal relapse, Image-guided interventional radiotherapy, Brachytherapy, Outcomes, Toxicity

## Abstract

**Background:**

This study aimed to evaluate clinical outcomes in patients with vaginal recurrences treated with radiotherapy with or without chemotherapy, followed by image-guided interventional radiotherapy (IG-IRT).

**Methods:**

We retrospectively analyzed patients with vaginal recurrences treated with external beam radiotherapy (EBRT) ± chemotherapy followed by endovaginal high dose rate IG-IRT. The EBRT total dose was 45 Gy. The interventional radiotherapy boost delivered 28 Gy in four high-dose-rate fractions, achieving 85–95 Gy EQD2 (α/β 10) to the high-risk clinical target volume and 60 Gy EQD2 (α/β 10) to the intermediate-risk clinical target volume. The primary endpoint was local control. Secondary endpoints included metastasis-free survival, overall survival, cancer-specific survival, and acute and late toxicities.

**Results:**

Thirty-two patients (median age, 54 years) were included. Two-year actuarial local control, metastasis-free survival, overall survival, and cancer-specific survival rates were 100%, 77.8%, 93%, and 93%, respectively. At univariate analysis, chemotherapy was significantly associated with a reduced risk of metastasis (*p* = 0.0038). Among chemotherapy regimens, cisplatin-based therapy showed the lowest metastatic risk, with metastases occurring in 5% of patients (*p* = 0.019). Age > 59 years was significantly associated with increased mortality (*p* = 0.026). At multivariate analysis, age was the only independent predictor of mortality (*p* < 0.05). Acute grade 2 gastrointestinal and skin toxicity occurred in two patients, while one patient experienced late grade 3 vaginal stenosis.

**Conclusions:**

Image-guided interventional radiotherapy as a boost following EBRT for vaginal cuff recurrences is an effective and safe treatment option, providing excellent local control with a favourable toxicity profile.

## Introduction

Vaginal recurrences from endometrial or cervical cancer remain among the most challenging scenarios in gynecologic oncology, particularly after surgery. In endometrial cancer, recurrences often develop at the site of the post-hysterectomy vaginal cuff but may also involve more extensive areas of the vaginal wall [1–4]. Patients with substantial lymphovascular space invasion or aggressive histologic subtypes, such as serous carcinoma, may present with multifocal relapse patterns, indicating a more complex disease course [5]. Prognosis largely depends on the initial risk classification, with low-grade endometrioid tumors associated with more favourable outcomes than high-grade or non-endometrioid cancers [1]. Recent evidence has also highlighted the prognostic role of molecular risk factors, including p53 mutations and mismatch repair deficiency [6–8]. In cervical cancer, central vaginal recurrences following radical hysterectomy represent a critical therapeutic challenge. Tumor size, depth of stromal invasion, lymphovascular space invasion, and non-squamous histology are well-established predictors of recurrence and aggressive disease biology [2, 3].

The main treatment options for recurrent disease are surgery and radiotherapy [9]. Surgical management of vaginal recurrences is particularly demanding; achieving negative margins often requires radical procedures such as pelvic exenteration, which are associated with significant morbidity and a profound impact on quality of life. As a result, organ-preserving strategies have become increasingly important.

External beam radiotherapy combined with image-guided interventional radiotherapy (modern brachytherapy) has emerged as a cornerstone approach, providing high local control while avoiding the morbidity associated with exenterative surgery [9]. The availability of CT- and MRI-compatible applicators allows accurate volumetric treatment planning and adaptive dose delivery [9, 10]. The aim of the present study was to evaluate the efficacy and safety of salvage radiotherapy with or without chemotherapy followed by interventional radiotherapy for vaginal relapse after surgery in patients with endometrial or cervical cancer.

## Material and methods

### Study design and endpoint

A retrospective analysis of 32 consecutive patients treated for recurrent endometrial and cervix cancer between January 2019 and July 2025 was performed. Eligibility criteria included: (1) recurrence confirmed by biopsy; (2) vaginal relapse without evidence of distant metastases; (3) salvage treatment involving radio-chemotherapy followed by interventional radiotherapy; (4) MRI availability, and (5) written informed consent. All procedures were conducted in accordance with institutional and national research committee standards, as well as the ethical principles outlined in the Declaration of Helsinki.

The primary endpoint of the study was local control rate. Secondary endpoints included metastasis-free survival, overall survival, cancer-specific survival, and the incidence and severity of both acute and late toxicities rates. Actuarial local control was calculated from the date of the end of definitive radiotherapy to the occurrence of disease relapse or progression within the treated radiotherapy field, or to the date of the last follow-up. Actuarial metastasis-free survival was measured from the date of the end of definitive radiotherapy to the date of disease progression outside the treated field or to the last follow-up. Actuarial overall survival was defined as the time interval between the date of the end of definitive radiotherapy and either death from any cause or the last follow-up. Cancer-specific survival was defined as the time from definitive radiotherapy to death attributable to the disease. Toxicities were graded according to the Common Terminology Criteria for Adverse Events, version 5.0. All patients received instructions regarding intimate cleansing and hydration and the use of vaginal dilators.

Patients were evaluated weekly during radiation therapy. Once treatment was completed, patients have planned surveillance every 3 months for the first 2 years (clinical examinations, MRI and complete blood count) and then every 6 months for the next 3 years for recurrence of disease or toxicity from therapy.

#### Procedures

This study was performed within the framework of the COnsortium for BRachytherapy Data Analysis initiative, following its standardized guidelines. Collected data encompassed patient demographics, histologic subtype, radiotherapy technical and dosimetric parameters, treatment-related toxicities (acute and late), follow-up duration, and clinical outcomes.

### External beam radiotherapy

External beam radiotherapy was delivered to a total dose of 45 Gy in 25 fractions using intensity-modulated or volumetric-modulated arc therapy. In patients with pelvic nodal involvement, a simultaneous integrated boost was delivered to achieve a total dose of 60 Gy. Concurrent chemotherapy consisted of weekly cisplatin (40 mg/m^2^).

### Interventional radiotherapy

All patients received endovaginal image-guided high-dose-rate interventional radiotherapy, delivering a total dose of 28 Gy over four high dose rate fractions in order to achieve a total equivalent dose in 2 Gy fractions (EQD2, α/β = 10) of 85–95 Gy to the high-risk clinical target volume and 60 Gy EQD2 to the intermediate-risk clinical target volume. The image guided high dose rate interventional radiotherapy treatment schedule consisted of delivering two sessions per day, separated by a minimum interval of six hours, twice weekly. For all patients, a multichannel applicator was used.

Magnetic resonance imaging was performed at the end of radio ± chemotherapy to assess the feasibility of image-guided interventional radiotherapy. After the image-guided interventional radiotherapy implantation, a second MRI was acquired for delineation of gross tumor volume, high-risk clinical target volume (HR-CTV) and intermediate-risk (IR) clinical target volume.

OncentraBrachy Treatment Planning System (Elekta, Sweden) was used to generate the four CT-based treatment. Total equivalent dose in 2 Gy fractions was calculated by combining contributions from both external beam radiotherapy and interventional radiotherapy, using an α/β ratio of 10 Gy for tumor tissue and 3 Gy for organs at risk.

Target volumes were delineated using a combination of MRI and CT in accordance with the guidelines of the Gynaecological Groupe Européen de Curiethérapie, the Advisory Committee on Radiation Oncology Practice, American Brachytherapy Society, and Canadian Brachytherapy Group Consensus [9]. Contouring included the gross tumor volume (GTV), HR-CTV, and IR-CTV. The GTV comprised all visible tumor at the time of image-guided interventional radiotherapy, as determined by clinical assessment and MRI. The HR-CTV included the residual tumor along with adjacent vaginal tissues. The IR-CTV covered the disease extent at initial diagnosis plus a 0.5-cm safety margin. In case of complete remission to radio ± chemotherapy only the intermediate-risk clinical target volume was contoured including the disease extent at initial diagnosis in the superior and inferior directions and the entire thickness of the vaginal wall. Bladder, rectum, and small bowel were considered as organ at risk and they were contoured and considered during planning. After applicator reconstruction, dwell positions and times were optimized manually to achieve the prescribed dose while respecting organ constraints. Dose limits were set at EQD2 = 90 Gy to 2 cc of bladder, and EQD2 = 75 Gy to 2 cc of the rectum, and small bowel.

### Analysis of data and statistical methods

Statistical analysis was performed using univariate and multivariate methods. Actuarial outcomes were estimated using the Kaplan–Meier method, with subgroup comparisons performed using the log-rank test. Associations between clinical variables and outcomes were evaluated using univariate analysis, and independent predictors were identified using multivariate logistic regression models.

## Results

### Patient, tumor, and treatment characteristics

The study enrolled 32 patients with vaginal recurrences from endometrial or cervical cancer between January 2019 and July 2025. Patient and lesion details are summarized in Table 1.

**Table 1:** Patient and tumor characteristic

No of patients	32
Median age	54 years old (range 40–80)
Median follow-up	17 months (range 4–73)
*Primary tumour*	
Cervix	21
Endometrium	11
*Site of vaginal relapse*	
Vaginal vault	25
Vaginal wall	7
*Concomitant chemotherapy*	
Weekly CDDP 40 mg/m^2^	24

CDDP: cisplatin; No: number

The median age at diagnosis was 54 years old (range 40–80). Most patients experienced relapse at the level of the vaginal vault (25 patients, 78%).

The median total external beam radiotherapy dose delivered to the pelvis was 45 Gy with the tumor receiving a median of 45 Gy and sometimes an overdosage up to 60 Gy. Five patients with pelvic nodal metastases received a boost with a nodal dose of 60 Gy EQD2. High-dose-rate image-guided interventional radiotherapy was delivered using the OncentraBrachy treatment planning system and a Flexitron (Elekta, Stockholm, Sweden) device with a 192Ir source. The median brachytherapy dose was 28 Gy (14–30 Gy) over four or six high dose rate fractions. Twenty-four patients underwent concurrent weekly cisplatin chemotherapy (40 mg/m^2^), administered as 2-h intravenous infusions up to 5–6 cycles.

In the plan sum (EBRT *plus* IGA-IRT), the median minimum dose delivered to the most irradiated 2 cubic centimeters of rectum, bladder, and small bowel was 64.9 Gy (range 38.4–72.5), 67.8 Gy (range 50.5–87.1) and 57 Gy (range 18.8–71.2 Gy), respectively.

### Treatment outcomes

**Fig. 1:**
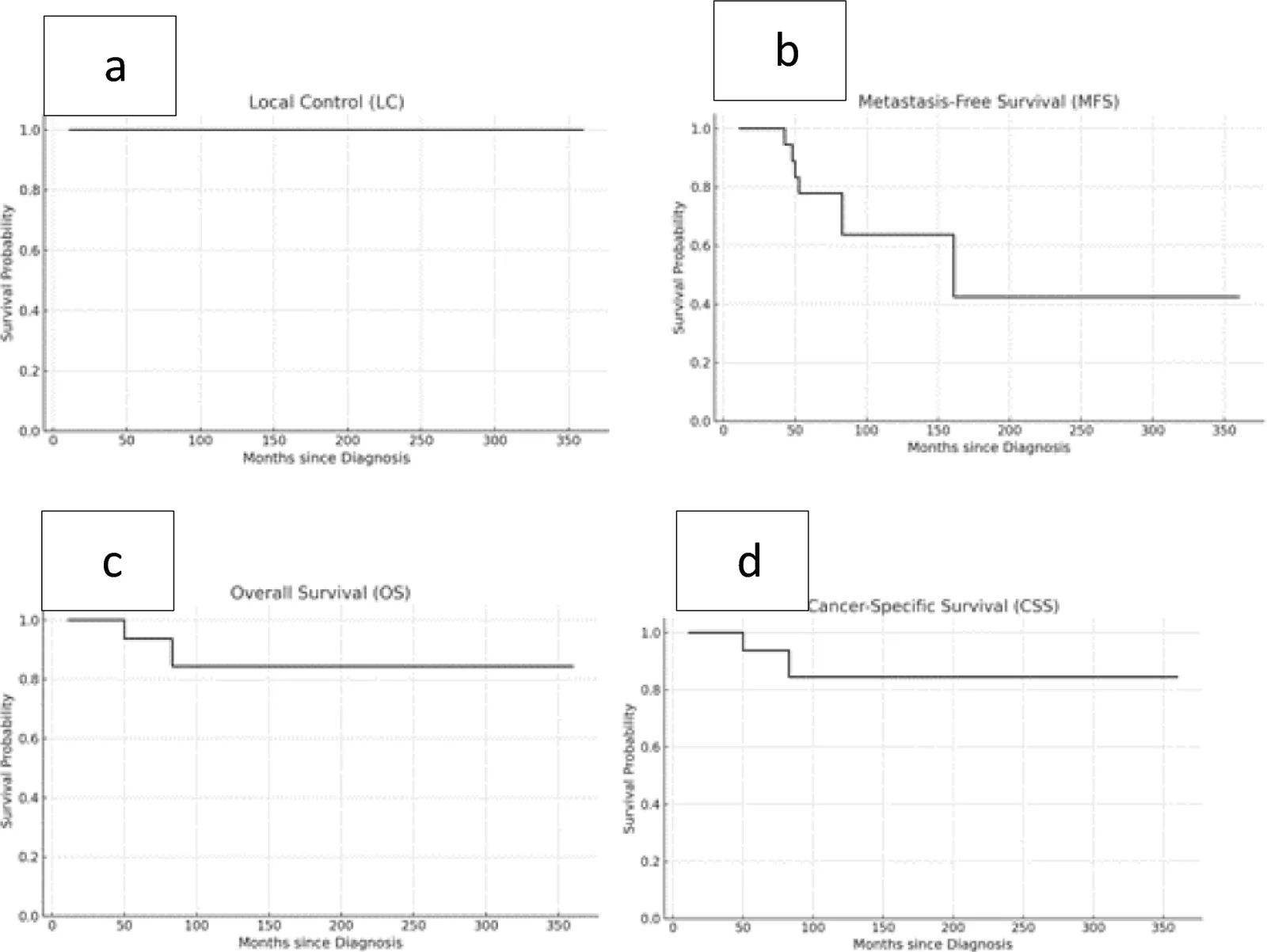
**a** 2-year actuarial local control rate; **b** 2-year metastasis-free survival rate; **c** 2-year overall survival rate; **d** 2-year cancer specific survival rate

No patients experienced relapse at the tumor site. Seven patients (21.8%) developed distant metastases and were treated with chemotherapy. The median time from the surgery to vaginal relapse was 26 months (range 9–329). With a median follow-up duration of 17 months (range 4–73), the 2-year actuarial local control rate was 100% (Fig. 1A), the 2-year metastasis-free survival rate was 77.8% (Fig. 1B), the 2-year overall survival rate was 93% (Fig. 1C) and the 2-year cancer specific survival rate was 93% (Fig. 1D).

The median tumor size reduction rate (RM at diagnosis-RM pre-interventional radiotherapy) was 97.9% (range 21.4%-100%). At univariate analysis, treatment with chemotherapy was significantly associated with low risk of metastasis (*p* = 0.0038). Among chemotherapy regimens, cisplatin-based regimen was associated with the lowest risk of metastasis, with only 5% of patients developing metastatic disease, compared with higher rates observed in other treatment groups (*p* = 0.019). Age > 59 years was significantly associated with a higher risk of death (*p* = 0.026). No statistically significant associations were observed between metastatic risk and age (*p* = 0.44), primary tumor type (*p* = 0.66), nodal status (*p* = 0.71), or percentage tumor reduction (*p* = 0.75). No significant associations were found between clinical status and chemotherapy (*p* = 0.24), type of chemotherapy (*p* = 0.60), nodal status (*p* = 0.22), primary tumor type (p = 0.88), or tumor reduction percentage (*p* = 0.70). At multivariate analysis, age emerged as the only independent predictor of death, with increasing age associated with higher odds of mortality after adjustment for all other covariates (adjusted OR > 1, *p* < 0.05). Patients aged 59 years or older showed a significantly increased risk of death compared with younger patients.

Regarding acute toxicity, no grade 4 events were observed. Most reported toxicities were grade 1 or 2. One case of grade 3 vaginal stenosis and one case of rectovaginal fistula were both observed 15 months after treatment. Further details on toxicity are provided in Table 2.

**Table 2:** Acute and late toxicities

Acute Toxicity	Grade				
	0	1	2	3	4
Diarrhea		1	1		
Asthenia		2	1		
Epidermolysis		1	1		
*Late toxicity*					
Fistula					1
Stenosis		1	2	1	
Proctitis			1		
Atrophy			4		

## Discussion

The present study demonstrates that salvage radiotherapy with or without chemotherapy followed by image-guided interventional radiotherapy provides excellent local control and favourable survival outcomes in patients with vaginal recurrence of endometrial or cervical cancer. At a median follow-up of 17 months, a 2-year local control rate of 100% was achieved, with limited acute and late toxicity, supporting the safety and efficacy of this approach.

These results are consistent with previously published series evaluating the role of interventional radiotherapy in the management of vaginal recurrences. A phase II study by Chopra et al. reported 5-year local control, disease-free survival, and overall survival rates of 84%, 73%, and 74.5%, respectively, in patients with postsurgical vaginal recurrence of cervical cancer treated with interventional radiotherapy. In that study, MRI-defined tumor volume > 20 cc was the only independent predictor of worse oncological outcomes [10]. Similarly, Engineer et al. reported favourable long-term outcomes with combined radiotherapy approaches, with 7-year local control and overall survival rates exceeding 65% [11]. In endometrial cancer, Sapienza et al. observed improved disease-free survival in patients treated with combined external beam radiotherapy and interventional radiotherapy, particularly in cases of isolated vaginal relapse [12].

The excellent local control observed in our cohort may be explained by the delivery of high total doses to the high-risk clinical target volume, with EQD2 values ranging between 85 and 95 Gy. Previous studies have demonstrated that dose escalation beyond 70 Gy EQD2 at the site of recurrence is associated with improved local control [13, 14]. In our experience, the integration of MRI and CT imaging throughout the treatment course enabled accurate target volume delineation and adaptive dose modulation according to tumor response, allowing for effective dose escalation while maintaining organ-at-risk constraints [15].

From a systemic disease perspective, chemotherapy was significantly associated with a reduced risk of distant metastasis at univariate analysis, particularly in patients receiving cisplatin-based regimens. This finding suggests a potential role of chemotherapy in controlling micrometastatic disease, although this benefit did not translate into improved overall survival [10, 12]. Conversely, age emerged as the only independent predictor of mortality in multivariate analysis, indicating that patient-related factors such as biological frailty and comorbidities may play a more relevant role in long-term survival than treatment-related variables in this setting [1, 8, 13, 16].

Regarding toxicity, despite the delivery of high doses to the target volumes, the incidence of severe late adverse events was low. Only One case of grade 3 vaginal stenosis and one case of rectovaginal fistula were both observed 15 months after treatment. This favourable toxicity profile compares well with previous reports, in which significant late toxicities such as proctitis and cystitis have been described [10–12, 17–19]. The low toxicity rates observed in our study may be attributed to meticulous image-guided planning, careful applicator selection, and adaptive dose optimization, as well as to multidisciplinary management and patient education, including the use of vaginal dilators and supportive care measures.

From a radiological and interventional perspective, image-guided interventional radiotherapy represents a paradigm shift toward personalized, procedure-driven oncologic treatment. The integration of MRI and CT imaging allows real-time adaptation of target volumes and dose distribution based on tumor response, reinforcing the central role of imaging not only in diagnosis and follow-up but also in treatment delivery and optimization [9, 10]. This approach highlights the evolving role of the radiologist and radiation oncologist as interventional specialists directly involved in therapeutic decision-making [9, 10, 14]. Clinically, these findings support the use of image-guided interventional radiotherapy as a definitive salvage strategy for vaginal recurrences, particularly when delivered in specialized centres with expertise in advanced image-guided procedures [9, 10, 20, 21]. Multidisciplinary tumor board discussions remain essential to appropriately select candidates for this approach and to tailor treatment according to individual patient and disease characteristics [22–27].

The main limitations of this study include its retrospective design, the relatively small sample size, and the limited follow-up duration, which may restrict the evaluation of long-term survival and late toxicity. Larger, multi-institutional prospective studies are warranted to further validate these results and to identify prognostic factors that may guide personalized treatment strategies.

## Conclusion

The present definitive approach shows promising oncological and toxicity outcomes for patients with vaginal recurrence. Developing an optimal therapeutic strategy for these patients requires careful multidisciplinary discussion. Moreover, randomized trials are needed to further guide clinical decision-making and enable truly personalized treatment plans for individuals with vaginal recurrence.
